# Patient Feedback on a Mobile Medication Adherence App for Buprenorphine and Naloxone: Closed and Open-Ended Survey on Feasibility and Acceptability

**DOI:** 10.2196/40437

**Published:** 2023-04-19

**Authors:** Crystal L Smith, Abigail Keever, Theresa Bowden, Katie Olson, Nicole Rodin, Michael G McDonell, John M Roll, Gillian Smoody, Jeff LeBrun, Andre QC Miguel, Sterling M McPherson

**Affiliations:** 1 Department of Community and Behavioral Health Elson S Floyd College of Medicine Washington State University Spokane, WA United States; 2 Analytics and PsychoPharmacology Laboratory Washington State University Spokane, WA United States; 3 Program of Excellence in Addictions Research Washington State University Spokane, WA United States; 4 College of Pharmacy and Pharmaceutical Sciences Washington State University Spokane, WA United States; 5 Ideal Option Spokane, WA United States; 6 Optimize Health Seattle, WA United States

**Keywords:** opioids, mobile technology, medication adherence, buprenorphine, naloxone, opioid use disorder, opioid agonist therapy, medication-assisted treatment, medications for opioid use disorder, opioid, opioid use, well-being, quality of life, clinic, technology, medication, remote, coaching, tracking, stress, incentive, tool, mobile phone

## Abstract

**Background:**

Opioid use disorders impact the health and well-being of millions of Americans. Buprenorphine and naloxone (BUP and NAL) can reduce opioid overdose deaths, decrease misuse, and improve quality of life. Unfortunately, poor medication adherence is a primary barrier to the long-term efficacy of BUP and NAL.

**Objective:**

We aimed to examine patient feedback on current and potential features of a Bluetooth-enabled pill bottle cap and associated mobile app for patients prescribed BUP and NAL for an opioid use disorder, and to solicit recommendations for improvement to effectively and appropriately tailor the technology for people in treatment for opioid use disorder.

**Methods:**

A convenience sample of patients at an opioid use disorder outpatient clinic were asked about medication adherence, opioid cravings, experience with technology, motivation for treatment, and their existent support system through a brief e-survey. Patients also provided detailed feedback on current features and features being considered for inclusion in a technology designed to increase medication adherence (eg, inclusion of a personal motivational factor, craving and stress tracking, incentives, and web-based coaching). Participants were asked to provide suggestions for improvement and considerations specifically applicable to people in treatment for opioid use disorder with BUP and NAL.

**Results:**

Twenty people with an opioid use disorder who were prescribed BUP and NAL participated (mean age 34, SD 8.67 years; 65% female; 80% White). Participants selected the most useful, second-most useful, and least useful features presented; 42.1% of them indicated that motivational reminders would be most useful, followed by craving and stress tracking (26.3%) and web-based support forums (21.1%). Every participant indicated that they had at least 1 strong motivating factor for staying in treatment, and half (n=10) indicated children as that factor. All participants indicated that they had, at some point in their lives, the most extreme craving a person could have; however, 42.1% indicated that they had no cravings in the last month. Most respondents (73.7%) stated that tracking cravings would be helpful. Most respondents (84.2%) also indicated that they believed reinforcers or prizes would help them achieve their treatment goals. Additionally, 94.7% of respondents approved of adherence tracking to accommodate this feature using smart packaging, and 78.9% of them approved of selfie videos of them taking their medication.

**Conclusions:**

Engaging patients taking treatment for opioid use disorder with BUP and NAL allowed us to identify preferences and considerations that are unique to this treatment area. As the technology developer of the pill cap and associated mobile app is able to take into consideration or integrate these preferences and suggestions, the smart cap and associated mobile app will become tailored to this population and more useful for them, which may encourage patient use of the smart cap and associated mobile app.

## Introduction

### Background

In 2019, a total of 10.1 million people aged 12 years and older reported misusing opioids, with 1.6 million of those meeting criteria for an opioid use disorder [1]. Intravenous opioid use increases the risk of contracting blood-borne illnesses such as hepatitis B, hepatitis C, and HIV [2]. Buprenorphine and naloxone (BUP and NAL) can reduce opioid overdose deaths by at least 70%, decrease opioid misuse, and improve quality of life [3-6]. Because of its demonstrated efficacy, the National Institute on Drug Abuse, the Substance Abuse and Mental Health Services Administration, the American Society of Addiction Medicine, and the American Academy of Addiction Psychiatry endorse the use of medications for opioid use disorder in the treatment of opioid use disorder (eg, BUP and NAL and methadone).

### Medication Adherence

One of the most substantial barriers to the long-term efficacy of BUP and NAL and poor medication adherence is consistently associated with relapse and overdose risk, as well as several other negative outcomes for patients [7,8]. In a study of 703 patients examining the effect of BUP adherence on opioid relapse, patients who were nonadherent to their prescribed BUP were over 10 times more likely to relapse than patients who were adherent [9]. Poor medication adherence can occur for myriad reasons, as summarized by the World Health Organization’s Five Dimensions of Adherence: social and economic, health care system, condition-related, therapy-related, and patient-related [10]. At the patient level, age, perception of treatment, co-occurring psychiatric conditions, and environment (eg, living with other substance users or at a distance from treatment centers) are common causes for treatment discontinuation [8,11-13]. For example, a previous study found that 73% of patients in treatment for an opioid use disorder, with co-occurring symptoms of depression, maintained abstinence at 6 months of treatment. Of those who had combined symptoms of depression and anxiety, only 40% were abstinent in the same time period [14]. Anxiety is a predictive factor for additional psychopathologies, such as insomnia and sleep deprivation, which can, in turn, manifest into symptoms of anxiety and depression [15]. Additionally, a poor therapeutic alliance between patient and provider and lack of prescription monitoring may contribute to patient nonadherence, in part due to limited tools at the level of the prescribing clinician [16]. Nonadherence with BUP and NAL is frequently associated with an increased risk of diversion [17-21]. Given that BUP and NAL is a schedule III opioid medication, improving the management of adherence is critical to decreasing relapse, diversion, and overdose.

### Pillsy Technology

Pillsy, which has successfully and significantly increased adherence in other treatment areas, acts like a digital medication coach, providing reminders using a mobile app, text messages, and automated phone calls [22]. The platform automatically tracks doses and timing and sends intelligent reminders to create a unique feedback loop, which allows the developers to constantly optimize incentive or reminder messages to meet user needs and increase adherence. The Pillsy technology was developed to be a simple, inexpensive adjunct to standard treatment. Unlike some other technologies, such as the *MySafeRx* system [23], the Pillsy system does not require codes or video calls for the patient to access their medication. Still, other technologies, such as reSET-O by Pear Therapeutics, have integrated an intensive behavioral intervention into their platform [24]. Pillsy was designed intentionally without these requirements in an effort not to increase the patient burden, and to begin work with people with opioid use disorder using a simple platform that could be adjusted to specifically include features that were requested by patients to increase the practical usability of the product.

Considering the evidence that tracking medication intakes and maintaining a high motivation with prompts are effective in the intervention phase of adherence up to persistence and considering that Pillsy only nominally increases the cost of oral BUP and NAL treatment and providers can bill for monitoring time (CPT code 99091), it is a potentially attractive solution to patients, providers, and payers. However, there are no studies available that have systematically investigated this technology’s impact on medication adherence in this population. The device is under investigation in a small (ClinicalTrials.gov NCT04656899, n=40, 12 weeks) clinical trial that recently completed data collection.

### This Study

The intent of this investigation was to examine patient feedback regarding the acceptability and feasibility of an inexpensive Bluetooth-enabled pill bottle cap and associated mobile app (ie, Pillsy, an Optimize Health product) designed to monitor and increase BUP and NAL adherence with the assistance of a designated “helper” (eg, friend and family member). We gathered feedback from patients in BUP and NAL outpatient treatment on existing and potential features of the technology as well to solicit recommendations for improvement in an effort to tailor the technology most effectively and appropriately for people in opioid use disorder treatment.

## Methods

This study was conducted in the US states of Idaho and Washington.

### Participants

Inclusion criteria were being 18 years of age or older and receiving treatment for a substance use disorder at Ideal Option outpatient addiction medicine clinics. Patients at this clinic are on opioid substitution therapy. Participants may have begun receiving treatment at this clinic due to iatrogenic opioid dependency or heroin or fentanyl or other illicit opioid use prior to treatment induction.

### Ethical Considerations

Study procedures were classified as exempt by the Washington State University institutional review board. Participants received a US $25 Walmart gift card (not usable for tobacco, alcohol, or firearms) for participating.

### Study Design

Participants were a convenience sample of people receiving medication-assisted treatment that were recruited through 2 Ideal Option clinics. Ideal Option is specialized in medication-assisted treatment of opioid use disorder. Clinic front desk staff invited patients to participate as they arrived at the clinics for their usual care appointments using a short script describing the study. All participants were invited while a study staff member was there. If a study staff member was not present at the clinic, people were not being invited to participate. All research was conducted in 1 appointment at the respective clinic in a private room. Hours for recruitment varied by day based on the researcher’s availability, and all data were gathered by 1 researcher. Recruitment was conducted from May 2021 through June 2021. Interested patients met with research staff at the clinic for an eligibility screening, consenting, and study completion in 1 session. Participants were not required to have a smartphone or computer to participate in this study. Researchers provided a laptop for participants to use to view screenshots of the Pillsy system and complete the survey for the study.

Survey questions were developed by the research team. Pilot testing on the items was conducted by members of the research lab conducting the study. All surveys were administered by the first author of the manuscript. Participants reported their demographics, medication adherence, diversion, opioid cravings, experience with technology, and support system, and provided suggestions on how to best support people taking BUP and NAL. This information was gathered through a series of closed and open-ended questions administered on the provided laptop, with free text boxes for open-ended questions. Researchers used scripts and screenshots to describe the Pillsy intervention and familiarize patients with it. Potential features under consideration were described verbally. Participants were asked to rate their interest in or opinion on current and potential features of the app, using response options such as “useful, second most useful, and least useful.” They were also asked to provide detailed feedback (eg, concerns and suggestions) on examples of product features being considered through open-text areas in the survey. Specific features evaluated included the inclusion of a personal motivational factor identified by the patient, craving tracking, web-based health coach access for the treatment of anxiety or stress, sleep and activity tracking, an online moderated forum, and incentives for optimal medication implementation. Data were collected using the Research Electronic Data Capture, a secure data collection tool. Participation in the study took approximately 30 minutes.

### Statistical Analysis

Survey responses were analyzed using descriptive statistics (response frequencies and percentages) to characterize participant behavioral patterns and feedback.

## Results

### Study Population

A total of 20 participants (N=20) were assessed for eligibility, and all met the inclusion criteria. One participant ended participation after demographics due to a childcare issue that required immediate presence, resulting in n=19 participants for the feedback portion of the study. Participant ages ranged from 20 to 53 (mean 34.15, SD 8.67) years. Participants were primarily White (80%, n=16), female (65%, n=13) and identified as women (65%, n=13). In total, 80% (n=16) of the sample indicated their race as White, and 95% (n=19) indicated non-Hispanic or Latino ethnicity. The mean number of weeks participants had been in treatment at Ideal Option was 1.64 years (SD 86.19 weeks; range 1-261 weeks). The average number of times participants had been in treatment for opioid use disorder was 2.42 (SD 1.95; range 1-7 times).

### Survey Responses

#### Medication Adherence, Treatment Motivation, and Diversion

Participants were asked whether they had a strong motivating factor for staying in treatment and what that motivational factor was (see Table 1). Each participant indicated that they had at least 1 strong motivating factor, and 10 indicated children as that factor. When asked how easy or difficult it was to remember to take their medication doses, the majority (79%, n=15) indicated “easy” or “very easy,” and 42% (n=8) indicated that they did not miss doses, even occasionally. For those who did occasionally miss doses, the most frequent reason indicated was forgetting (88%, n=7), though people also indicated that they occasionally missed doses due to sleeping through the time when they were supposed to take it, being embarrassed to take it in public, or being in too much of a hurry.

**Table 1:** Survey questions, response options, and descriptive results.

Question and response options				Participants
**Medication adherence, treatment motivation, and diversion**				
	**Some people have 1 really strong motivating factor, like wanting to climb a mountain or maintain custody of their children. If you had to choose 1 motivational factor, what would it be? n (%)**			
		Children	10 (53)	
		Other family members	4 (21)	
		Reasons related to self	8 (42)	
	**How easy or difficult is it for you to remember to take your medication doses? n (%)**			
		Very easy	6 (32)	
		Easy	9 (47)	
		Not easy or difficult	2 (10.5)	
		Difficult	2 (10.5)	
		Very difficult	0 (0)	
	**Do you ever miss any doses? n (%)**			
		Yes	11 (58)	
	**Have you ever been asked to give your opioid use disorder medication to somebody else? n (%)**			
		Yes	4 (21.1)	
	**Did you provide it to them? (Note: Only asked among those who responded yes to the above question.) n (%)**			
		Yes	2 (50)	
	**Have you ever asked anybody else for some of their opioid use disorder medication? n (%)**			
		Yes	9 (47.4)	
	**How would you rate your cravings at the worst point in your life? (4=worst) mean (SD)**			
		0-4	4 (0)	
	**How would you rate your cravings in the last month? (4=worst) mean (SD)**			
		0-4	1.11 (1.15)	
	**Does your opioid use disorder medication help with that craving? n (%)**			
		Yes	19 (100)	
	**How would you rate your worst craving within a few hours after taking your opioid 1 use disorder medication? (4=worst) mean (SD)**			
		0-4	0.95 (1.03)	
	**How would you rate your worst craving at any time after receiving your prescription 1 for opioid use disorder medication? (4=worst) mean (SD)**			
		0-4	1.94 (1.26)	
**Motivational reminders; cravings, anxiety, and stress tracking; and professionally moderated forum**				
	**How helpful do you think reminders about your motivating factor would be? (0=not at all helpful, 100=very helpful) mean (SD)**			
		0-100	84.74 (19.75)	
	**Do you have any concerns about this idea? n (%)**			
		Yes	3 (16)	
	**Do you think tracking cravings would be helpful? n (%)**			
		Yes	14 (74)	
	**Do you have any concerns about this idea? n (%)**			
		Yes	6 (32)	
	**Do you feel like you have a lot of anxiety or stress in your life? n (%)**			
		Yes	16 (84)	
	**Do you currently or have you ever received professional treatment for anxiety or stress? n (%)**			
		Yes	14 (74)	
	**Would you be interested in treatment for stress or anxiety if it was available to you on the internet or in an app? n (%)**			
		Yes	15 (79)	
**Sleep**				
	**How many hours per night do you sleep on average? mean (SD)**			
		Hours	6.84 (2.46)	
	**Do you currently track sleep quality on a device or app on your phone? n (%)**			
		Yes	17 (85)	
	**Would you be interested in using something like that [referring to sleep tracking], if it was available to you? n (%)**			
		Yes	16 (84)	
	**Do you think that understanding your sleep patterns would help you understand if you should be taking your opioid use disorder medication? n (%)**			
		Yes	15 (79)	
	**Would you be willing to share your sleep data with a professional if they could use it to help with your opioid use disorder? n (%)**			
		Yes	19 (100)	
	**Would this help you achieve your treatment goals? n (%)**			
		Yes	16 (84)	

#### Motivational Reminder Feature Feedback

Participants were asked to rate the helpfulness of a feature that would remind participants of their self-selected motivational factor when it is time to take a dose; 84.2% (n=16) of respondents did not have concerns about this feature. Concerns that were raised by participants included that this may make people feel guilty if they were not reaching their treatment goals and that it may trigger people to use illicit substances if the motivational factor was upsetting. One participant suggested that this may be more helpful for people who were “stronger in their sobriety.” Suggestions for improving this feature included diversifying ways to deliver motivation such as adding reinforcing complements when people were doing well, incorporating personalized motivational phrases that users could create, adding a picture of the motivational factor, adding a voice recording of the technology user saying the motivational factor, and including a counseling component such as letting the user know that they are loved and not being judged or bringing in a spiritual component and other positive factors in the person’s life.

#### Technology Use, Craving, Anxiety, and Stress Tracking

All participants had smartphones with unlimited texting and had used Bluetooth connectivity previously. The average length of time they had owned their current smartphone was 16.6 (SD 13.17; range 1-48) months.

Most participants had “a lot” of anxiety or stress in their lives (84.2%, n=16), the majority of which (73.7%, n=14) had received professional treatment for it. All participants were asked if they would be interested in a web-based treatment for anxiety or stress hosted by a real health coach, and 78.9% (n=15) affirmed that this would be helpful. All participants indicated that they had experienced “the most extreme craving a person could have” at the worst point in their life. In response to a question asking participants what their worst cravings were in the past month, 42.1% (n=8) indicated no craving, 57.9% (n=11) indicated that they had some craving, and 0% (n=0) indicated that they had “the most extreme craving a person could have.” Most respondents stated that tracking cravings would be helpful (73.7%, n=14) and 68.4% (n=13) did not have concerns with this idea. While there were comments regarding concerns with confidentiality, concerns about tracking cravings primarily focused on triggering a relapse.

Triggers, focusing on that makes the person think of needles and triggers. May be better to focus on positive instead of the negative. E.g., how are my positive thoughts today. What are my goals.

Participant 2

Might trigger people. Maybe start that feature when people are more solid in the program and have less chance of relapsing.

Participant 4

Would probably be helpful but could trigger people. You don't want to get triggered because you are thinking about it too much.

Participant 20

Participants provided suggestions for improvement of the craving tracking feature. These suggestions focused on two primary areas: (1) identifying triggers of the craving and (2) providing resources to the person experiencing the craving.

Journaling about it so you can look back and reflect when you have the next cravings.

Participant 1

Connect to provider that way the provider can call them if they are struggling and let them know they care and offer to see them.

Participant 4

Include notes so you can say triggers to the craving.

Participant 6

If the person is having cravings provide resources - e.g., support group or AA meetings etc.

Participant 14

Add what you are doing at the moment.

Participant 19

#### Reinforcers

Participants were queried on including reinforcers (ie, “rewards”) for consistently taking their opioid use disorder medication. Most participants (84.2%, n=16) believed that reinforcers would help them achieve their treatment goals. When asked about preferences for reinforcers, the majority endorsed a US $25 Walmart card as their top of 3 choices (73.7%, n=14) over a US $25 Amazon card (21.1%, n=4 endorsed) or a month of free cell phone minutes and data (5.3%, n=1 endorsed). After researchers explained that a feature such as this may necessitate adherence tracking (a feature that is currently a primary component of the Pillsy system using pill cap opening data), participants were asked whether they would be open to tracking their doses (94.7%, n=18 responded “yes”), using smart packaging (94.7%, n=18 responded “yes”), and taking a selfie video while taking medication doses (78.9%, n=15 responded “yes”).

#### Feature Preferences

Of the potential features reviewed, participants indicated that motivational reminders would be most useful (42.1%, n=8), followed by craving and stress tracking (26.3%), and web-based support forums (21.1%, n=4; see Table 2 for details). When asked why they selected the features they did, responses for motivational reminders focused on having reminders in general, reminding oneself why they are in treatment, and having reassurance in their choice to continue treatment.

Help remind you why you are doing this and to see that you are making progress toward why you want to do it.

Participant 2

Reminding me to take the meds and remember why I'm in it would be helpful.

Participant 14

**Table 2:** Application features rated by usefulness (N=19).

	Most useful, n (%)	Second most useful, n (%)	Least useful, n (%)
Motivational reminders	8 (42.1)	4 (21.1)	0 (0)
Craving and stress tracking	5 (26.3)	4 (21.1)	2 (10.5)
Online support forums	4 (21.1)	1 (5.3)	0 (0)
Reinforcers	2 (10.5)	5 (26.3)	2 (10.5)
Adherence tracking	0 (0)	2 (10.5)	1 (5.3)
Sleep tracking	0 (0)	3 (15.8)	3 (15.8)

Participants who selected craving and stress tracking as a feature preference explained that they would prefer the feature to increase awareness of triggers and make supports aware of a potential need for help.

If you are tracking cravings you can see what is causing it.

Participant 10

Tracking craving and stress would make people more aware and could cue others to help.

Participant 4

Additional feedback was solicited from participants on how to support people taking BUP and NAL. Although these were all garnered from the same open-ended free-text box, we have organized the feedback into the areas of medical providers and researchers, patients in medications for opioid use disorder programs, and improving treatment or technology. This feedback is listed in Table 3.

**Table 3:** Participant feedback quotes regarding how to support people taking BUP and NAL^a^.

Participant quotes			Participant number
**Medical providers and researchers**			
	Compassion. Don't assume drug addicts are stupid. Don't insult my intelligence. Treat people with respect no matter what level they are at in life or education. *Rewards feels like giving a dog a bone. I would be insulted.	15	
	*The rewards could be dangerous. People can sell gift cards and use the money to buy drugs and people will absolutely do that. I also think that people who really care about getting better don't need to be bribed to do it.*	3	
**Patients in MOUD^b^ programs**			
	*Be honest with yourself. Remember the reason you are doing this / getting treatment. If you aren't honest with yourself, you won't get the proper treatment you deserve.*	18	
	*Take it how it's prescribed. I tried splitting it up and it didn't work as well. I should have done it how it was prescribed in the first place. Also - I like having to come in every 2 weeks - the check ins hold me accountable, and I think less people will fail this way.*	17	
	*It's hard to tell people about relapse b/c I am prideful and get embarrassed. For people like me - don't be pushy asking about things like that. I will share when I am ready. It's hard when people are pushy.*	10	
	*Once you start getting through to where you have been on treatment for a while and it doesn't make you high anymore (people say Suboxone doesn't get you high, but it does) don't give up on it. In a few months don't get off it just because you feel normal - that's the goal - to just feel normal and function like a normal person.*	7	
	*Try to get yourself happy. Help create stability. Distract the users with positivity or vacation. Get them out of their situation, their life, providing fun things to help them forget about their addiction and shitty life. Take them out of the shittiness and they will want to help themselves.*	5	
**Improving treatment or technology**			
	*Have a feature where someone would get ahold of the person if they were having a hard time. Bring them closer to keep them on the right track.*	4	
	*More interaction is better because it gives people less room to screw up. Tell people ‘Remember who you are doing it for.’ Use personal quotes and 1 liner motivational messages.*	3	
	*I have been disappointed that there haven't been groups available. If I had a community, I wouldn't be so isolated (I am very isolated).*	2	
	*Messaging option where you can contact a provider / hotline or email or text messaging for quick answers (counselor).*	1	

^a^BUP and NAL: buprenorphine and naloxone.

^b^MOUD: medications for opioid use disorder.

## Discussion

### Principal Findings

This descriptive study examined feedback from a sample of patients in opioid use disorder treatment to better understand their perceptions and opinions of how a Bluetooth-enabled pill bottle cap and the associated mobile app could be used to improve adherence to opioid use disorder treatment. Poor medication adherence can be the result of discord between patient and provider, lack of resources (availability of transportation to a clinic), and mental health challenges, such as anxiety. Participants indicated that they had strong motivating factors for wanting to stay in treatment. Personalized reminders that target incentives for sobriety, and avoid self-identified triggers for anxiety and cravings, could prove useful in improving the Pillsy technology. Feedback was overwhelmingly positive regarding a feature that would remind them of their self-selected motivational factor when it is time to take a medication dose. A feature providing reinforcers for consistently taking opioid use disorder medications was endorsed at greater than 80%. In addition, over 70% of participants indicated that tracking cravings would be helpful.

The results of this study support modifications to the Pillsy technology that would integrate additional motivational interventions to help promote medication adherence in people with opioid use disorder. First, strong support was present for the integration of personalized motivational factors, follow-up by the research team on suggestions made by participants to include phrases of encouragement and pictures of the family will be considered. Second, the integration of contingency management to provide tangible reinforcers for medication adherence will be considered, particularly, considering the efficacy of contingency management interventions in substance use treatment [25].

Our sample did not suggest additional safeguards such as required video check-ins or unique codes from their provider be added to the technology. This will continue to differentiate the Pillsy technology from other medication access technologies, such as *MySafeRx*. Our sample also did not request educational materials or behavioral interventions similar to those in the reSET-O technology, therefore continuing to differentiate Pillsy from Pear Therapeutics technology.

### Limitations

The study has limitations that should be acknowledged. First, the sample for the study was a small (N=20) convenience sample that was geographically homogeneous (all participants received care within Idaho and Washington states in the United States), primarily White and largely female. Selection bias may be present, as participants were people who had time to engage in the study following their scheduled visit to the treatment clinic. Second, while the participants were given descriptions of how the technology being investigated functioned, they did not have the opportunity to interact with it.

### Conclusions

Providing treatment adherence promotion through a digital Bluetooth-based smart pill cap and associated mobile app, like Pillsy, maybe a way to increase adherence and improve treatment outcomes for people in treatment for opioid use disorder using BUP and NAL. Including patient feedback and preferences in the development process is integral to creating a user-friendly, meaningful intervention that patients will use and find helpful. The valuable feedback provided through this study will allow the technology developers to modify the app based on this feedback, with the intention of creating a more patient-centered product.

## Abbreviations

BUP: buprenorphine
NAL: naloxone

## Data Availability

Data are available upon request, pending approval of the study’s primary investigator and external funding sponsor for the project.
